# Seasonality of tuberculosis in intermediate endemicity setting dominated by reactivation diseases in Hong Kong

**DOI:** 10.1038/s41598-021-99651-9

**Published:** 2021-10-12

**Authors:** Leonia Hiu Wan Lau, Ngai Sze Wong, Chi Chiu Leung, Chi Kuen Chan, Alexis K. H. Lau, Linwei Tian, Shui Shan Lee

**Affiliations:** 1 Jockey Club School of Public Health and Primary Care, The Chinese University of Hong Kong, Hong Kong , China; 2 Stanley Ho Centre for Emerging Infectious Diseases, The Chinese University of Hong Kong, Hong Kong, China; 3 Hong Kong Tuberculosis, Chest and Heart Disease Association, Hong Kong, China; 4 Tuberculosis and Chest Service, Centre for Health Protection, Department of Health, Hong Kong, China; 5 Department of Civil and Environmental Engineering, The Hong Kong University of Science and Technology, Hong Kong, China; 6 Division of Environment and Sustainability, The Hong Kong University of Science and Technology, Hong Kong, China; 7 School of Public Health, Li Ka Shing Faculty of Medicine, The University of Hong Kong, Hong Kong Special Administrative Region, China

**Keywords:** Epidemiology, Tuberculosis

## Abstract

Summer-spring predominance of tuberculosis (TB) has been widely reported. The relative contributions of exogenous recent infection versus endogenous reactivation to such seasonality remains poorly understood. Monthly TB notifications data between 2005 and 2017 in Hong Kong involving 64,386 cases (41% aged ≥ 65; male-to-female ratio 1.74:1) were examined for the timing, amplitude, and predictability of variation of seasonality. The observed seasonal variabilities were correlated with demographics and clinical presentations, using wavelet analysis coupled with dynamic generalised linear regression models. Overall, TB notifications peaked annually in June and July. No significant annual seasonality was demonstrated for children aged ≤ 14 irrespective of gender. The strongest seasonality was detected in the elderly (≥ 65) among males, while seasonal pattern was more prominent in the middle-aged (45–64) and adults (30–44) among females. The stronger TB seasonality among older adults in Hong Kong suggested that the pattern has been contributed largely by reactivation diseases precipitated by defective immunity whereas seasonal variation of recent infection was uncommon.

## Introduction

Spring–summer predominance of tuberculosis (TB) incidence has been widely reported across countries and populations^1^, contrary to the recognised winter season for other respiratory infections (e.g., influenza)^2^. While seasonal pattern of acute respiratory infections is ascribed directly to variation in transmission dynamics^3^, mechanisms underlying the seasonal nature of TB are far more complex. The relatively long and variable incubation period of TB could dampen the seasonal variability of the observed TB incidence caused by mycobacterial transmission. Furthermore, population incidence of TB is contributed by not just exogenous recent infection but also endogenous reactivation of latent tuberculosis infection (LTBI)^3^, the summative effects of which could be hard to delineate by season.

Several mechanisms for the spring–summer surges of TB incidence have been hypothesised. One dominant hypothesis suggested increased transmission in wintertime, probably due to indoor congregation^4,5^ and/or seasonal variation in meteorological factors favouring mycobacterium survival^6^. Separately, relative immunosuppression in winter due to seasonal fluctuation of vitamin D level^4,7^, air pollution^8,9^ and/or co-infection with seasonal respiratory virus^10^ were also shown to be at play, which could affect either disease progression from recent infection or reactivation of LTBI. From a population perspective, a fundamental question on TB seasonality is the relative contribution of any of these hypothesised mechanisms and the balance between recent infection and endogenous reactivation in the disease burden. Delineating the variations in seasonal patterns of TB among population subgroups might shed light on this subject, but the reported results were inconclusive^3,11–16^.

**Figure 1:**
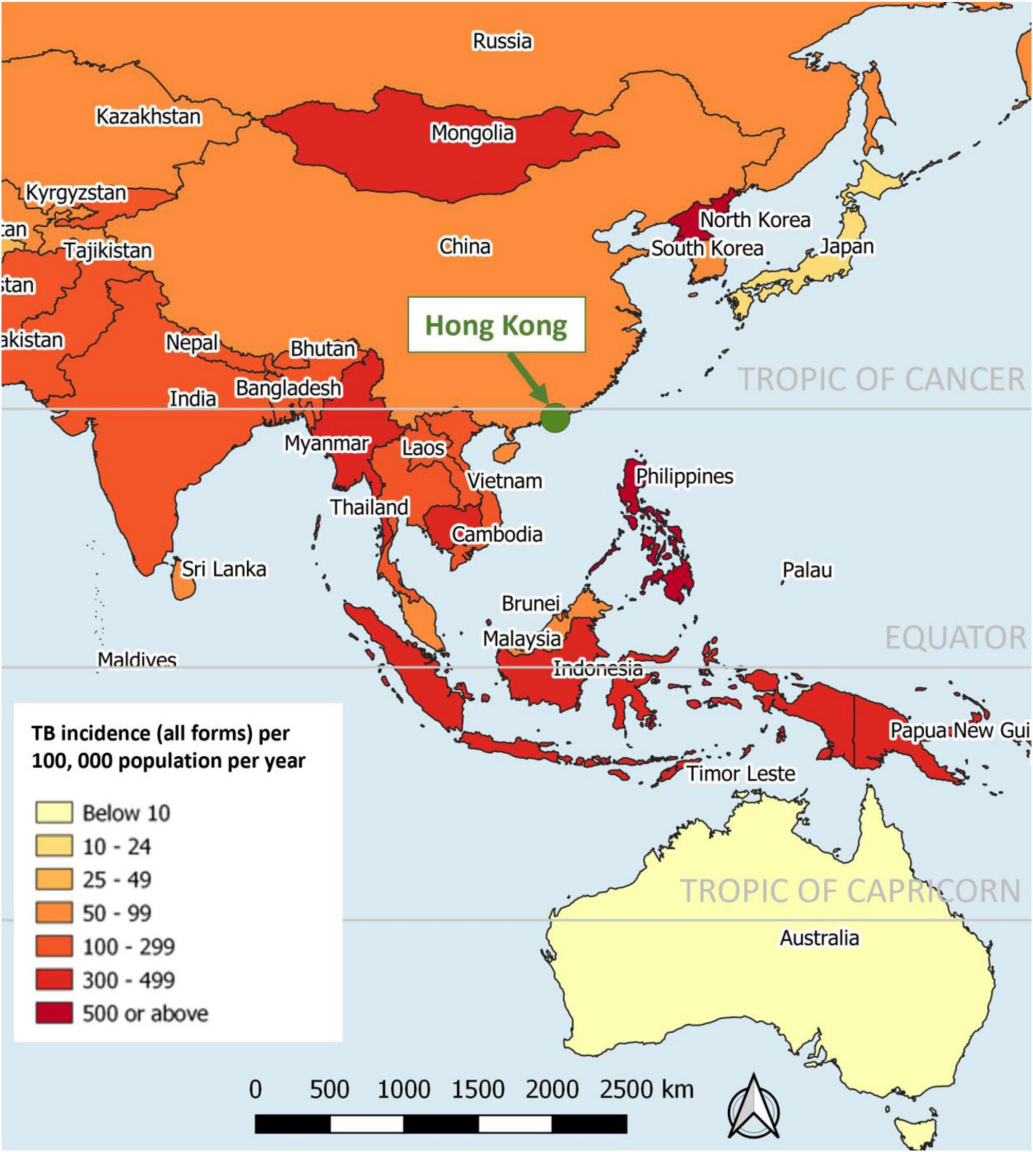
Tuberculosis incidence in the Asia–Pacific region, 2017 (per 100,000 population). We downloaded the country boundaries from GADM database (https://gadm.org/data.html), and the global TB incidence data from world Health Organization (https://www.who.int/data/gho/data/themes/tuberculosis) for the generation of this figure.

Worldwide, TB epidemiology in both high- and low-burden countries are largely attributed to mycobacterial transmission, either directly within the population or as a result of migration from higher TB burden countries respectively, while endogenous reactivation plays a relatively less important role in driving the epidemics. In Asia Pacific, Hong Kong is a metropolitan city with intermediate TB burden driven primarily by endogenous reactivations^17,18^. Located on southern coast of China (Fig. 1), Hong Kong has an oceanic subtropical monsoon climate which is characterised by hot and humid summer from May through August (average temperature 27–31 °C; average relative humidity 83%), and cool to mild winter between November and February (average temperature 14–18 °C; average humidity 70%) (https://www.hko.gov.hk/en/cis/climahk.htm). Aging population and successful control of tuberculosis transmission prompted a shift of incidence towards older-age groups (from 21% of overall TB notifications in 1990 to around 44% in 2018)^19^, while increased proportion of which arose from reactivation of past infection (from about 50% in mid 1960s to more than 80% in late 1990s, and almost to 100% by 2015)^17,20^. It is speculative that the relative contributions of exogenous recent infection and endogenous reactivation to the observed TB seasonality may vary greatly between settings with different TB burden. Furthermore, sharing the non-stationary properties of epidemiological time series, seasonal patterns of TB incidence/notification time series tend to be time-varying. Fluctuations in magnitude and shifts in timing of periodic surge in disease incidence over time have complicated the modelling of TB seasonality, while classical stationary approaches assuming constant seasonal pattern might not be justified. Against such background, we set out to investigate the TB seasonality in Hong Kong resulting largely from endogenous reactivation, focusing on the application of non-stationary approaches to characterise the seasonal patterns of TB notifications in population subgroups defined by age, gender, and disease forms.

## Materials and method

### Data source and case definition

TB is a statutorily notifiable disease in Hong Kong, the reporting of which from clinical services in public and private sectors are centrally collated in the Department of Health. Notification data for all forms of active TB cases between 2005 and 2017, covering the socio-demographic (gender; age; ethnicity; place of birth and residency status), clinical (case category: new cases or retreatment/relapsed cases; disease forms: pulmonary with or without extrapulmonary involvement, or extrapulmonary only; sputum smear and culture status) and epidemiological (date of notification; risk factors and comorbidities) characteristics, were retrieved from the territory-wide TB registry maintained by the Department of Health. An active TB case was defined as disease confirmed by positive isolation of *Mycobacterium tuberculosis* complex or, in the case of absent bacteriological confirmation, disease diagnosed on clinical, radiological, and/or histological grounds together with an appropriate response to anti-TB treatment.

### Ethics

Ethical approval was obtained from The Joint Chinese University of Hong Kong -New Territories East Cluster Clinical Research Ethics Committee (The Joint CUHK-NTEC CREC). Informed consent was waived by The Joint CUHK-NTEC CREC as the collected data were anonymised and accessed retrospectively. The dataset cannot be included in a public repository because the data are owned by third parties. Access to these data and permission could be inquired through the Department of Health, Hong Kong SAR Government. All methods were performed in accordance with the relevant guidelines and regulations.

### Data analyses

The daily notification data were aggregated into monthly counts. In view of the age—^21^, gender—^22^ and disease form—^23^ differential in epidemiological burden of TB, the aggregated notification data were first stratified by age (0–14 children; 15–29 young adults; 30–44 adults; 45–64 middle aged; and $\ge$ 65 elderly) and gender (male; female), then further evaluated according to disease forms (pulmonary TB with or without extrapulmonary involvement; extrapulmonary TB only). We defined TB seasonality as systematic, repetitive, periodic variation in its notifications with calendar time, the patterns of which is characterised by the seasonal amplitude (peak-to-tough ratio) and peak timing^24^. Considering the possibility of non-stationary properties of time series of TB notification, the seasonal parameters of which were potentially time-varying, the degree of seasonality was defined as a combined effect of (1) seasonal amplitude and (2) predictability of seasonal variation (degree of consistency in seasonal amplitude and peak timing)^25^. Wavelet analysis coupled with dynamic generalised linear regression models (DGLMs) were used to examine the seasonality of TB notifications among different population subgroups.

In wavelet analysis, we applied continuous wavelet transforms (CWT) to the monthly counts of TB notifications for each subgroup of interest, to examine the periodicity of each time series^26^. We first constructed the global wavelet power spectrum to determine the presence of an overall dominant and significant annual periodicity averaged throughout the time series^27^. Second, we analysed the time evolutions of the annual periodic components with local wavelet power spectrum, to reveal the degree of seasonality regarding the predictability of seasonal variation (i.e., proportion of time-steps over the full time series with significant power at the annual scale)^27^. DGLMs were then fitted to the time series for which wavelet analysis could detect a significant annual seasonality dominating throughout the study period, to further quantify the degree of seasonality in terms of peak-to-trough ratio and identify the peak timing of the seasonality^28,29^ (Supplementary Text for full technical details). Sensitivity analysis was conducted by applying the wavelet transform and fitting the regression model to subgroups stratified by various cut-off age. Statistical analyses were performed using R 4.0.0, with the “KFAS” package employed for development of DGLMs^30^.

## Results

### Basic characteristics of the notified TB cases in Hong Kong

There were altogether 64,386 active TB cases in the notification registry during the observation period (from 2005 to 2017), ranging over 156 months. The male-to-female ratio was 1.74:1. Elderly patients aged $\ge$ 65 accounted for more than one-third (41%) of the overall TB notifications, which was about 60 times that for children less than 15 years old (0.7%) (Table 1; Supplementary Table 1). A majority of notified TB cases were permanent Hong Kong residents (93%). More than 60% were born outside Hong Kong. Pulmonary TB was diagnosed in 85% of patients. Monthly standardised notification rate (SNR) of pulmonary TB gave a decreasing trend for all age-gender subgroups, while increasing trends were observed among extrapulmonary cases (Supplementary Figure 1). Co-morbid illness was reported in 27% of all TB notifications (Table 1), which was more common among males (31% in males versus 20% in females) and the elderly ≥ 65 (39% in the elderly ≥ 65 versus 18% among those < 65). Diabetes was the most frequently reported co-morbid condition. Silicosis, lung cancer and other chronic respiratory disease appeared to be male dominant TB comorbidities, while autoimmune diseases appeared to be more prevalent among female TB patients.

**Table 1:** Profile (socio-demographic and clinical) of notified TB cases in Hong Kong by age, gender, and disease form, 2005–2017.

	Male, n = 40,908; no. (%)				Female, n = 23,478; no. (%)			
	Pulmonary TB^a^		Extrapulmonary TB		Pulmonary TB^a^		Extrapulmonary TB	
	Aged ≥ 65	Aged < 65	Aged ≥ 65	Aged < 65	Aged ≥ 65	Aged < 65	Aged ≥ 65	Aged < 65
Total no. of TB notifications	17,619	19,222	1666	2401	5765	12,061	1338	4314
**Place of birth**								
Hong Kong	3207 (19.0)	10,835 (58.6)	318 (19.8)	1472 (63.1)	1092 (19.8)	5643 (48.6)	311 (23.80)	1848 (43.8)
Mainland China and Macau	13,290 (78.8)	6900 (37.3)	1227 (76.3)	674 (28.9)	4263 (77.4)	3585 (30.9)	933 (71.5)	1229 (29.2)
Other key Asian countries^b^	241 (1.4)	594 (3.2)	44 (2.7)	154 (6.6)	111 (2.0)	2292 (19.7)	44 (3.4)	1114 (26.4)
Miscellaneous	125 (0.7)	160 (0.9)	20 (1.2)	33 (1.4)	44 (0.8)	98 (0.8)	17 (1.3)	24 (0.6)
**Permanent HK residency**^**c**^								
Yes	17,370 (99.7)	18,469 (97.1)	1629 (99.5)	2271 (96.4)	5666 (99.5)	9449 (79.1)	1309 (99.6)	3201 (75.6)
No	48 (0.3)	546 (2.9)	8 (0.5)	85 (3.6)	30 (0.5)	2497 (20.9)	5 (0.4)	1034 (24.4)
**Employment status**								
Full time employment	629 (3.6)	10,259 (53.9)	77 (4.7)	1367 (57.8)	52 (0.9)	6265 (52.3)	25 (1.9)	2345 (54.9)
Students	3 (0.02)	1121 (5.90	1 (0.1)	163 (6.9)	2 (0.03)	970 (8.1)	1 (0.1)	178 (4.2)
Homemaker	18 (0.1)	4 (0.02)	4 (0.2)	3 (0.1)	3892 (68.1)	2977 (24.9)	796 (60.6)	1176 (27.5)
Retired/unemployed	16,496 (94.4)	6781 (35.6)	1522 (92.6)	739 (31.2)	1622 (28.4)	1273 (10.6)	469 (35.7)	451 (10.6)
Others	337 (1.9)	886 (4.7)	40 (2.4)	93 (3.9)	148 (2.6)	491 (4.1)	23 (1.8)	122 (2.9)
**Case category**								
New cases	14,711 (84.0)	17,304 (90.5)	1493 (91.1)	2207 (93.0)	5200 (90.9)	11,364 (94.7)	1252 (94.8)	4013 (93.8)
Previous infected OR treated cases	2792 (16.0)	1824 (9.5)	146 (8.9)	167 (7.0)	523 (9.1)	635 (5.3)	69 (5.2)	263 (6.2)
**Sputum smear status**								
Smear positive	6105 (36.3)	7706 (42.2)	0 (0)	0 (0)	1700 (31.8)	4237 (37.4)	0 (0)	0 (0)
Smear negative	10,717 (63.7)	10,551 (57.8)	1029 (100.0)	1599 (100.0)	3639 (68.2)	7090 (62.6)	723 (100.0)	2854 (100.0)
**Culture confirmed cases (sputum)**								
Culture positive	13,588 (84.2)	13,075 (74.6)	0 (0)	0 (0)	4131 (80.6)	7590 (70.1)	0 (0)	0 (0)
Culture negative	2557 (15.8)	4459 (25.4)	847 (100)	1395 (100)	993 (19.4)	3239 (29.9)	621 (100)	2560 (100)
**Drug resistance cases**^**d**^ ***(among those with culture positive status)***								
Drug resistance	53 (0.4)	154 (1.2)	0 (0)	0 (0)	8 (0.2)	86 (1.1)	0 (0)	0 (0)
**Co-morbid illness**								
Presence of any co-morbid condition	7051 (40.0)	4486 (23.3)	695 (41.7)	399 (16.6)	2157 (37.4)	1483 (12.3)	509 (38.0)	1347 (31.2)
Diabetes mellitus	3048 (17.3)	2831 (14.7)	299 (17.9)	170 (7.1)	1050 (18.2)	736 (6.1)	217 (16.2)	147 (3.4)
Lung cancer	539 (3.1)	229 (1.2)	26 (1.6)	16 (0.7)	108 (1.9)	68 (0.6)	16 (1.2)	14 (0.3)
Other malignancies	1117 (6.3)	631 (3.3)	151 (9.1)	91 (3.8)	280 (4.9)	221 (1.8)	135 (10.1)	133 (3.1)
Chronic renal failure	258 (1.5)	151 (0.8)	74 (4.4)	54 (2.2)	75 (1.3)	59 (0.5)	49 (3.7)	53 (1.2)
HIV infection	33 (0.2)	199 (1.0)	5 (0.3)	40 (1.7)	3 (0.1)	49 (0.4)	0 (0.0)	13 (0.3)
Silicosis and other pneumoconiosis (e.g., asbestosis)	155 (0.9)	130 (0.7)	11 (0.7)	7 (0.3)	2 (0.03)	2 (0.02)	0 (0.0)	1 (0.02)
Others chronic respiratory disease^e^	1441 (8.2)	327 (1.7)	70 (4.2)	20 (0.8)	242 (4.2)	135 (1.1)	20 (1.5)	19 (0.4)
Gastrectomy	114 (0.6)	32 (0.2)	17 (1.0)	1 (0.04)	20 (0.3)	3 (0.02)	3 (0.2)	2 (0.05)
General debilitation (due to old age, immobility, stroke)	1966 (11.2)	77 (0.4)	214 (12.8)	6 (0.2)	762 (13.2)	26 (0.2)	162 (12.1)	13 (0.3)
Autoimmune disease^f^	113 (0.6)	138 (0.7)	21 (1.3)	24 (1.0)	69 (1.2)	193 (1.6)	28 (2.1)	64 (1.5)
Received organ transplantation	8 (0.05)	27 (0.1)	0 (0.0)	10 (0.4)	1 (0.02)	26 (0.2)	0 (0.0)	3 (0.1)
Liver disease^g^	76 (0.4)	78 (0.4)	9 (0.5)	12 (0.5)	14 (0.2)	19 (0.2)	6 (0.4)	6 (0.1)
On cytotoxic/steroid/biologics or other immunosuppressant	165 (0.9)	180 (0.9)	20 (1.2)	39 (1.6)	79 (1.4)	235 (1.9)	24 (1.8)	54 (1.3)

^a^Patients classified as pulmonary TB may include extrapulmonary TB.

^b^Including Philippines, Indonesia, Thailand, Nepal, Vietnam, India, Pakistan, and Bangladesh.

^c^7 years of stay in Hong Kong required for permanent residency, except by birth.

^d^Resistance to at least one first line antituberculosis agent.

^e^Including chronic obstructive pulmonary disease (COPD); bronchiectasis; asthma; interstitial lung disease.

^f^Including systemic lupus erythematosus; Sjogren’s syndrome; multiple sclerosis; myasthenia gravis; Guillain–Barre Syndrome; ankylosing spondylitis; rheumatoid arthritis; psoriatic arthritis. Grave’s disease; Addison’s disease; Hashimoto’s thyroiditis; ulcerative colitis; Crohn’s disease, primary biliary cirrhosis; celiac disease; psoriasis; autoimmune blistering disease (e.g., pemphigus; pemphigoid; Ig-mediated bullous dermatoses); autoimmune encephalitis (e.g., acute disseminated encephalomyelitis); sarcoidosis; vitiligo; antiphospholipid; pernicious anaemia; aplastic anaemia; idiopathic thrombocytopenia purpura; autoimmune vasculitis (e.g., Behcet's syndrome; Giant cell arteritis; Churg-Strauss syndrome; Takayasu’s arteritis; polymyalgia rheumatic); Glomerulonephritis; IgA nephropathy; Goodpasture’s syndrome; Wegener’s granulomatosis; scleroderma; polymyositis; dermatomyositis.

^g^Including fatty liver; liver cirrhosis; liver fibrosis.

### Degree and timing of male TB seasonality by age group and form of disease

All forms of TB notifications in male elderly (aged $\ge$ 65) was dominated by significant seasonal oscillation in annual cycles between 2005 and 2012 (Fig. 2a). After 2012, periodicity pattern with bi-annual frequency, despite insignificant, had emerged. Further quantification of seasonal amplitude with regression model revealed a decreasing peak-to-trough ratio from 24 to 14% (Table 2). By TB disease form, comparable seasonal pattern was observed for the pulmonary but not extrapulmonary cases (Table 3; Fig. 2c,e).

**﻿Figure 2:**
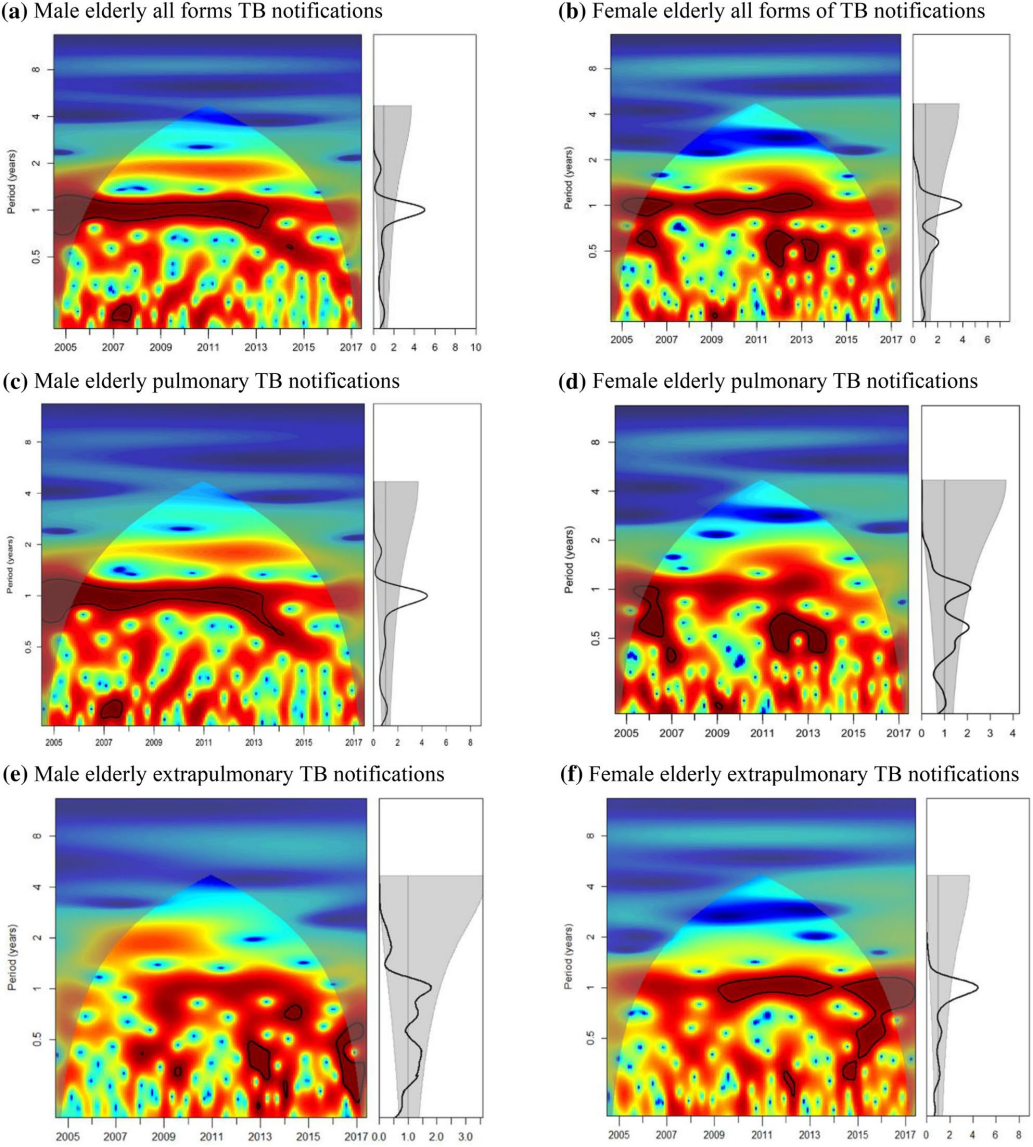
Wavelet analysis of monthly notification of elderly (≥ 65) TB in Hong Kong from 2005–2017, for (**a**) males’ all forms TB cases; (**b**) females’ all forms TB cases; (**c**) males’ pulmonary TB cases; (**d**) females’ pulmonary TB cases; (**e**) males; extrapulmonary TB cases; and (**f**) females’ extrapulmonary TB cases. (Right) Global wavelet power spectrum: thick black lines represent the global wavelet power estimates and the grey band indicate the 95% confidence bounds against red-noise background spectra. Significant annual periodicity is indicated when the peak of power exceeds the grey band at 1-year period. (Left) Local wavelet power spectrum: Wavelet power value is shown in colour from dark blue (low value) to dark red (high value). Area enclosed with black contour lines indicates the 5% significance level against red noise. Lighter shade area indicates the cone of influence where the edge effect becomes important, and the spectral information is less robust.

**Table 2:** Seasonality of all forms of tuberculosis by gender and age.

Age group	Gender	Continuous wavelet transform (CWT)^a^		Dynamic generalised linear regression (DGLMs)			
		Global power spectrum: (i.e., presence of dominant annual periodicity)	Local power spectrum: (predictability of seasonal variation: time evolution of annual periodicity)	Seasonal amplitude (peak-to-tough ratio)		Peak timing in year (months)	
				Maximum (95% CI)	Minimum (95% CI)	Maximum (95% CI)	Minimum (95% CI)
Elderly (≥ 65)	Male	Annual seasonality	Strongly evident from 2005–2012	1.24 (1.15–1.30)	1.14 (1.13–1.23)	6.53 (5.96–6.79)	6.32 (5.88–6.76)
			Weaker and non-significant after 2012				
	Female	Annual seasonality	Strongly evident from 2005–2006; 2008–2012	1.25 (1.16–1.33)	1.25 (1.17–1.35)	7.40 (6.97–7.99)	7.40 (6.94–8.00)
			Weaker and non-significant after 2012				
Middle aged (45–64)	Male	Annual seasonality	Strongly evident in 2005–2008 and 2012	1.16 (1.09–1.27)	1.16 (1.08–1.25)	6.67 (6.14–7.69)	6.67 (5.81–7.39)
			Weaker, non-significant in other time periods				
	Female	Annual seasonality	Strongly evident from 2005 and 2008–2014	1.30 (1.23–1.40)	1.30 (1.23–1.40)	7.24 (6.87–7.64)	7.24 (6.87–7.66)
			Weaker and non-significant after 2014				
Adults (30–44)	Male	No significant seasonality	–	–	–	–	–
	Female	Annual seasonality	Strongly evident in 2005–2013	1.29 (1.22–1.37)	1.29 (1.22–1.37)	6.58 (6.16–6.89)	6.57 (6.16–6.89)
			Weaker and non-significant after 2013				
Young adults (15–29)	Male	Annual seasonality	Strongly evident in 2005–2007; 2010–2013	1.20 (1.12–1.31)	1.20 (1.13–1.32)	7.13 (6.31–7.92)	7.13 (6.01–7.91)
			Weaker, non-significant in other time periods				
	Female	Annual seasonality	Strongly evident in 2005–2007	1.28 (1.17–1.38)	1.28 (1.17–1.39)	6.83 (6.23–7.44)	6.83 (6.25–7.44)
			Weaker, non-significant in other time periods				
Children (0–14)	Male	No significant seasonality	–	–	–	–	–
	Female	No significant seasonality	–	–	–	–	–

^a^Statistical significance of computed wavelet power spectrum was tested against the red noise background representing the null hypothesis that the observed time-series is no different to that expected from a purely random process, with significance level of 95%.

**Table 3:** Seasonality of pulmonary (PTB) and extrapulmonary tuberculosis (EPTB) by gender and age.

Age group	Gender	Pulmonary Tuberculosis						Extrapulmonary Tuberculosis					
		Continuous wavelet transform (power spectrum)		Dynamic generalised linear regression				Continuous wavelet transform (power spectrum)		Dynamic generalised linear regression			
		Global^a^	Local^b^	Seasonal amplitude (peak-to-tough ratio)		Peak timing in year (months)		Global^a^	Local^b^	Seasonal amplitude (peak-to-tough ratio)		Peak timing (months)	
Elderly (≥ 65)	Male	*	2005–2012	1.22 (1.13–1.33)	1.16 (1.12–1.24)	6.42 (5.96–6.82)	6.31 (5.85–6.80)	–	–	–	–	–	–
	Female	–	–	–	–	–	–	*	2009–2012; & 2014–2017	1.64 (1.50–2.22)	1.64 (1.45–1.91)	7.56 (7.11–8.09)	7.56 (6.97–8.01)
Middle aged (45–64)	Male	*	2005–2008; & 2012	1.16 (1.08–1.28)	1.16 (1.08–1.24)	6.65 (5.94–7.56)	6.65 (5.84–7.35)	*	2013–2015	1.33 (1.07–1.52)	1.22 (1.10–1.67)	7.52 (5.01–8.20)	5.44 (5.06–8.11)
	Female	*	2005–2013	1.33 (1.27–1.58)	1.33 (1.24–1.48)	7.50 (6.93–8.11)	7.49 (7.01–8.06)	*	2012–2015	1.28 (1.17–1.44)	1.28 (1.18–1.45)	6.60 (5.87–7.33)	6.60 (5.90–7.33)
Adult (30–44)	Male	–	–	–	–	–	–	–	–	–	–	–	–
	Female	*	2005–2007; &2011–2014	1.29 (1.20–1.40)	1.29 (1.20–1.39)	6.91 (6.33–7.37)	6.91 (6.34–7.36)	*	2005–2008	1.93 (1.13–2.10)	1.24 (1.09–1.59)	7.03 (4.17–7.13)	4.39 (3.93–7.66)
Youngadults (15–29)	Male	*	2005–2006; and 2013	1.18 (1.10–1.29)	1.18 (1.10–1.30)	7.14 (6.22–7.89)	7.14 (6.15–7.93)	*	2007–2008; and 2010–2013	1.48 (1.25–1.90)	1.22 (1.18–1.90)	8.14 (5.98–8.41)	5.79 (4.75–8.11)
	Female	*	2005–2007	1.28 (1.16–1.38)	1.28 (1.16–1.38)	7.13 (6.43–7.74)	7.13 (6.45–7.74)	*	2005; 2009	1.39 (1.16–1.72)	1.34 (1.15–1.69)	5.82 (4.75–6.90)	5.80 (4.62–7.00)
Children (0–14)	Male	–	–	–	–	–	–	–	–	–	–	–	–
	Female	–	–	–	–	–	–	–	–	–	–	–	–

^a^Global wavelet power spectrums determined the existence of a dominating and significant annual periodicity overall through the time series.

^b^Local wavelet power spectrums showed the time evolution of annual periodic components and predictability of the seasonal variation.

*Indicated presence of significant annual seasonality.

The relatively strong annual seasonality (i.e., higher predictability of seasonal variation and peak-to-trough ratio as compared with other age groups) was also detected in young adults (aged 15–29) group, for all forms of TB (Table 2) and extrapulmonary cases, but was less remarkable in pulmonary cases (Table 3). For the male middle aged (aged 45–64) group, an overall significant yet weaker annual periodicity was detected in all forms of TB notifications (Table 2). Among adults aged 30–44 and children (aged $\le$ 14) groups, no significant annual seasonality was demonstrated overall and for all forms of TB (Tables 2 and 3). Supplementary Figure 2 summarises all wavelet power spectra for different population subgroups.

As regards the seasonal timing, the highest peak months were between June and July for all forms of TB and pulmonary TB notifications for all age groups with significant annual seasonality detected. On the contrary, the peak timing in extrapulmonary cases was less concentrated, shifting from late May to August (Tables 2 and 3).

### Degree and timing of female TB seasonality by age group and form of disease

Throughout the time series, a significant annual periodicity was detected as the dominant scale of variation in all forms of TB notifications among the female elderly. Such annual periodicity was strongly evident from 2005 to 2006 and 2008 to 2012, with the peak-to-trough ratio fluctuating at around 25% (Fig. 2b; Table 2). Further stratification by forms of disease demonstrated a significant and prominent annual periodicity in extrapulmonary cases (significant annual pattern exhibited from 2009–2012 and 2014–2017; peak-to-trough ratio 64%) but not in pulmonary cases (Fig. 2d,f; Table 3).

Stronger annual periodicity of notifications for all forms of TB was exhibited among patients in the middle-aged group (significant annual pattern in 2005 and 2008 to 2014; peak-to-trough ratio 30%), and to a similar extent, among the adult groups (significant annual from 2005 to 2012; peak-to-trough ratio 29%) (Table 2). For the middle-aged group, further TB disease form stratification revealed prominent annual seasonality from 2005 to 2013 in pulmonary cases, and from 2012 to 2015 in extrapulmonary cases. On the other hand, the observed seasonality of notifications for all forms of TB among adult groups were mainly contributed by the pulmonary cases. An overall significant yet weaker annual periodicity was detected in all forms of TB notifications among the young adults group. No significant annual seasonality was demonstrated in children (aged ≤ 14) group (Tables 2 and 3). The peak timing of notifications in the female groups was between late June and July for all forms of TB and pulmonary cases, while the extrapulmonary cases tended to be less concentrated with shifting from late April to August (Tables 2 and 3).

Sensitivity analyses with shifts of 5-years age band detected similar patterns of seasonal variation in the TB notifications as in the primary analyses, indicating that the current age-stratified cut-offs was robust to characterise the variations of seasonal patterns across different population subgroups.

## Discussion

The present study showed that TB notifications in intermediate endemicity city Hong Kong were dominated by an annual seasonal pattern with peak timing in late spring to summer seasons, consistent with what has been reported previously^3^. The enhanced sensitivity of wavelet analysis allowed us to detect the degree of consistency among the time-varying seasonal parameters, while DGLMs allow parameters quantification through maximum likelihood estimation over a sliding time window. Characteristically, the annual seasonal patterns were found to vary across population subgroups as differentiated by age, gender, and disease forms. Overall, there was clearly no significant annual seasonality for TB notifications among children aged ≤ 14 years irrespective of gender and disease forms. For male groups aged ≥ 15 years, the strongest seasonality was detected among the elderly, and to a lesser extent, among the young adults. Gender-related difference in TB seasonality was observed. Stronger seasonality was consistently observed among females, with clearer pattern compared to males of the same age groups. In females, the seasonal variation was more prominent among the middle-aged and adults instead of the elderly for males.

Temporally, the highest peaks of monthly notification for all forms of TB and pulmonary TB were concentrated in June and July, corresponding with late spring to summer of the year in Hong Kong. Given the possibility of a 6 months’ lag period as reported previously to account for the slow progression of disease and reporting delays^3^, the pattern suggested the existence of seasonal factors related to increased propensity for disease development or transmission risks during wintertime. Increased transmission risk in winter has been widely proposed in high- (e.g., China, India) and low- (e.g., United Status) burden settings as the dominant driver of TB seasonality, as evidenced by demonstration of highest seasonal variation in children who had recently contracted TB^11–13^. Childhood TB has long been recognised as a direct consequence of adult TB and an indicator of recent transmission from adults in the community^31^. In our study, however, we found no significant seasonality in TB notifications among children aged ≤ 14 years. The discrepancy might be related to the small size of childhood TB population in Hong Kong. Our results did not coincide with the previous findings in an earlier cohort spanning 1991 to 2002, when there was relatively high incidence that ranged from 117 to 98 per 100,000 population^3^, from which large seasonal variation was detected among children under age 15. Our results were more similar to that of a recent study from Japan (with the lowest incidence among those intermediate burden settings; 17 per 100,000 population)^15^. Shifting dominance from mycobacterial transmission to endogenous reactivation in TB epidemiology of Hong Kong over the past decades could likely have accounted for such observations^17,20^.

Overall, strong seasonality of TB notifications in Hong Kong was exhibited in older adults including male aged ≥ 65 and females aged 45–64 years. Increased rate of reactivation of LTBI has been reported among the elderly in low- and intermediate-burden settings as a result of waning immunity with age^32,33^. In Hong Kong, nearly all TB cases among the elderly could be attributable to reactivation^17,20^. Parallelly, reactivation of latent infection also accounted for a majority of TB cases among young adults in Hong Kong^34^, which is attributed to the intermediate endemicity setting characterised by a low exposure risk in the community. Taken together, the TB seasonality demonstrated in our study has more likely resulted from reactivation of latent infection, rather than disease due recent infections. The immunological rhythm appeared to be exaggerated at older age, owing to the immunosenescence and the underlying chronic illnesses which may further compromise immunity^35^. With a population ageing, the predominance of TB diseases due to reactivation would likely continue in Hong Kong and countries with similar epidemiology.

Rising age is one of the causes of relative immune deficiency, underlying the notable seasonality demonstrated for reactivation TB. Seasonal immune modulation of autoimmune disease (e.g., rheumatoid arthritis)^36^, as evidenced by cyclical fluctuation of immune responses^37,38^, has been increasingly reported. As extrapulmonary TB disease was more common among immunocompromised hosts^39^, their dominance lent further support to the contribution of LTBI reactivation to the observed TB seasonality. Separately, environmental factors such as vitamin D and air pollutants could display seasonal patterns, the presence of which could drive the seasonality of TB reactivation. In this connection, Vitamin D is a known modulator of antimicrobial capacities of macrophages and T-cell function^40^. Exposure to air pollutants such as PM_10_ and PM_2.5_ was shown to result in overladen alveolar macrophages with impaired function^41^. Furthermore, periodic particulate matter (PM) exposure could modulate the balance between Th1 and Th2 immune response against mycobacteria, thereby varying the risk of TB reactivation^42,43^. Immunosuppression and subsequence TB reactivation might also be induced by co-infection with viral or bacterial pathogens exhibiting winter seasonality (e.g., influenza)^13^. Overall, the varied occurrence of these environmental and microbiological factors on TB reactivation could explain the variability of its seasonality among adults in Hong Kong.

The gender-related difference in TB seasonality could be probably explained by the role of sex hormone, particularly the estrogens^44^. Estrogens are known immune modulator, influencing Th1/Th2 immune balance and playing a major role in those female preponderant autoimmune diseases, e.g., systemic lupus erythematosus (SLE)^45^. TB reactivation is associated with a shift from Th1 to Th2 cytokines profile, and a predominant Th2 profile in female population has been reported^46^, which may account for the stronger TB seasonality observed in females, particularly for the middle-aged groups. The weaker seasonality detected in female young adults is puzzling, but might be associated with the gender-related difference in BCG cross-reactivity^34^. Furthermore, increased predominance of extrapulmonary TB in driving the TB seasonality was observed in middle aged, and to a greater extend, in the elderly. Previous study reported that older women were less able to contain mycobacterium in lungs due to dramatic reduction of sex hormones after menopause^47^. Interestingly, while strong seasonality was detected in pulmonary cases among the male elderly, we found that such seasonality was absent among the female elderly. Existence of a strong non-seasonal risk factors such as in-door pollution could be a reason for the observations^48^.

We acknowledge that this study did carry some limitations. Owing to the ecological nature of study design, the results were insufficient on their own to lead to a definitive conclusion regarding the underlying reasons of TB seasonality. Nevertheless, using the surveillance data of large sample size accrued over a long period of time, our findings have provided evidence of the existence of seasonality resulting from TB reactivation in an intermediate TB burden setting. There is limited epidemiological evidence to delineate the differential effects of host, environmental and microbiological factors on TB reactivation. Further investigations, including correlation of the TB reactivation and the seasonal immunomodulating factors such as vitamin D, air pollutant and respiratory viral infection, are warranted to establish the mechanism of TB seasonality.

## Conclusion

TB notifications exhibits stronger seasonality among older adults, who accounted for the majority of TB cases in Hong Kong. The observations suggested that the TB seasonality in Hong Kong has been contributed largely by reactivation diseases precipitated by defective immunity in older adults, whereas seasonal variation of recent TB infection was uncommon. The analyses drew attention to potentially modifiable risk factors associated with TB seasonality, which could inform the development of preventive and control measures.

## Supplementary Information

Supplementary Information.
