# Supplementary material for: Seasonality of tuberculosis in intermediate endemicity setting dominated by reactivation diseases in Hong Kong
Source: Sci Rep. 2021 Oct 12;11:20259. doi: 10.1038/s41598-021-99651-9 (PMC8511215; doi:10.1038/s41598-021-99651-9)
Supplement: Supplementary file 1 — Supplementary Information. [file 41598_2021_99651_MOESM1_ESM.docx]

**Seasonality of tuberculosis in intermediate endemicity setting dominated by reactivation diseases in Hong Kong**

**Supplementary material**

**Supplementary Text**

**I. Continuous wavelet transform (CWT)**

Continuous wavelet transforms (CWT) reveal the seasonal patterns of a given time series by decomposing the time series *x(t)* into time-frequency space, from which its spectral characteristics are estimated as a function of time [1].

The wavelet transform is achieved by convolutions of the time series with repeated computations of the cross-correlations (i.e., ‘local similarity’) between time series *x(t)* and a set of scaled and translated wavelets: $W_{x}\left( a, \tau\right)= \frac{1}{\sqrt{a}}\int_{-\infty}^{\infty} x\left( t \right)\psi^{*}(\frac{(t-\tau}{a})dt=\int_{-\phi}^{\phi} x\left( t \right)\psi_{a,\tau}^{*}\left( t \right)dt$ (where * denotes the complex conjugate form. Parameters $a$ and $\tau$ denote the dilation (scale factor) and translation (time shift), respectively) [1]. The wavelet function with particular scale is firstly compared with a section at the beginning of the time series, and the cross-correlation between the wavelet and that sections of the time series is calculated as the wavelet coefficients, $W_{x}\left( a, \tau\right)$. The wavelet is then shift along the time series, with the wavelet coefficients calculated for successive windows within the time series. After the convolutions for the first scale are complete, the wavelet is then scaled (i.e., ‘dilated’) so that it represents a longer period than the previous wavelet, the wavelet coefficients is calculated repeatedly for the new scale. This process is repeated for multiple scales, until the window length approaches the half of the length of whole time series. Morlet mother-wavelet was used as wavelet base function regarding of its good balance between time and frequency localisation [1]. Time series were detrended and normalized before applying transformation. We specified the initial scale of wavelet functions as 2 months, twice as long as the sampling interval, and set the spacing between scales at 1/250 (250 sub-octaves per octave).

Wavelet coefficients represent the contributions of various wavelets with specific scales *a* to the time series *x(t)* at particular time positions$\tau$ [2]. When the matching between the time series and wavelet is high, a higher positive value of coefficient would be given. The matrix of wavelet coefficients (i.e., output of CWT) could be used to estimate the local and global wavelet power spectrum. The dominant periodic components of the time series and its significance could be identified with the global wavelet power spectrum, the time-averaged representations of energy distribution of time series at different frequencies [2]. The time evolutions of the dominant periodic components (e.g., the annual scale), thus the strength/ degree of such periodicity, could be recovered by local wavelet power spectrum [2]. Statistical significance of computed wavelet power spectrum was tested against the red noise background representing the null hypothesis that the observed time-series is no different to that expected from a purely random process, with significance level of 95% [1,2].

**2. Dynamic generalised linear models (DGLMs)**

For the time series which the wavelet analysis detected an overall significant annual seasonality, we fitted the following dynamic generalised linear models to further quantify the degree of seasonality in terms of peak-to-trough ratio and identify the peak timing of the seasonality.

DGLMS is a dynamic extension of classic generalised linear model (GLMs), allowing the regression coefficients to change gradually over time in a random manner, with capacity to model the time-varying seasonal pattern in non-stationary time series. Denoting the TB notifications counts at time $t_{k}$ by $\left\{ y_{k} | k=1,\ldots\ldots,n \right\}$, which follow Poisson distribution with expected number of events $\lambda_{k}$, we fitted the model as follows [3,4]:

$\log\left( \lambda_{k} \right)=S_{k}+T_{k}$

$S_{k}=a_{k}sin( 2\pi{\cdot t}_{k}/12)+b_{k}\cos(2\pi\cdot t_{k}/12)$

$T_{k}= T_{k-1}+(t_{k}-t_{k-1})\cdot\alpha_{k}$,

where$S_{k}$ describes the seasonal components and $T_{k}$ describes the secular trend; $t_{k}$ represents the running index for month; $a_{jk}$, $b_{jk}$ and $\alpha_{k}$ are regression coefficients to be estimated from the data, which were allowed to change over time following a simple random walk [3,4]:

$a_{k}= a_{(k-1)}+ \varepsilon_{k}$

$b_{k}= b_{(k-1)}+ v_{k}$

$\alpha_{k}= \alpha_{k-1}+\omega_{k}$

where $\varepsilon_{k}$, $v_{k}$ and $\omega_{k}$ are independent variables following normal distribution with mean zero, variance $\Delta t_{k}W$ (for $\varepsilon_{k}$ and $v_{k}$) and $\Delta t_{k}V$ (for $\omega_{k}$). Given the dynamic nature of DGLMs, time-varying peak-to-trough ratio (${P/T ratio}_{k}=exp(2\gamma_{k})$; where $\gamma_{k}=\sqrt{{a_{k}}^{2}+{b_{k}}^{2}}$ ) and peak timing (${PeakTime}_{k}=12(1-\psi_{k}/\pi)/2;$ *where* $\psi_{k}= -arctan(a_{k}/b_{k}$)) could be extracted based on the estimated regression coefficients [4,5].

Confidence intervals (95%) of the seasonal parameters were obtained with the bootstrap percentile method, in which the sampling distributions of the seasonal parameters were constructed by fitting the regression model to 1000 datasets resampled from the original time series using moving blocks bootstrap.

**Supplementary Table 1. Basic Characteristics of the TB notified cases by year in Hong Kong, 2005 -2017**

| **Year** | **2005** | **2006** | **2007** | **2008** | **2009** | **2010** | **2011** | **2012** | **2013** | **2014** | **2015** | **2016** | **2017** | **Total** |
| --- | --- | --- | --- | --- | --- | --- | --- | --- | --- | --- | --- | --- | --- | --- |
| **Total no. of TB cases**  **(TB SNR per 100000 population)** | 6087  (89.3) | 5698  (83.1) | 5382  (77.7) | 5586  (80.1) | 5159  (74.0) | 5023  (71.5) | 4682  (66.2) | 4787  (66.9) | 4599  (64.0) | 4622  (63.9) | 4333  (59.4) | 4274  (58.3) | 4154  (56.2) | 64386  (69.8) |
| **Gender** | | | | | | | | | | | | | | |
| Male (%) | 3911  (64.3) | 3706  (65.0) | 3435  (63.8) | 3580  (64.1) | 3232  (62.6) | 3176  (63.2) | 3008  (64.2) | 3037  (63.4) | 2864  (62.3) | 2945  (63.7) | 2759  (63.7) | 2649  (62.0) | 2606  (62.7) | 40908  (63.5) |
| Female (%) | 2176  (35.7) | 1992  (35.0) | 1947  (36.2) | 2006  (35.9) | 1927  (37.4) | 1847  (36.8) | 1674  (35.8) | 1750  (36.6) | 1735  (37.7) | 1677  (36.3) | 1574  (36.3) | 1625  (38.0) | 1548  (37.3) | 23478  (36.5) |
| **Age** | | | | | | | | | | | | | | |
| 0-14 (%) | 61  (1.0) | 48  (0.8) | 48  (0.9) | 48  (0.9) | 44  (0.9) | 43  (0.9) | 33  (0.7) | 26  (0.5) | 32  (0.7) | 17  (0.4) | 24  (0.6) | 23  (0.5) | 25  (0.6) | 472  (0.7) |
| 15-29 (%) | 900  (14.8) | 776  (13.6) | 692  (12.9) | 720  (12.9) | 684  (13.3) | 627  (12.5) | 605  (12.9) | 567  (11.8) | 591  (12.9) | 502  (10.9) | 489  (11.3) | 457  (10.7) | 437  (10.5) | 8047  (12.5) |
| 30-44 (%) | 1110  (18.2) | 1025  (18.0) | 916  (17.0) | 934  (16.7) | 845  (16.4) | 831  (16.5) | 743  (15.9) | 826  (17.3) | 778  (16.9) | 730  (15.8) | 697  (16.1) | 672  (15.7) | 612  (14.7) | 10719  (16.6) |
| 45-64 (%) | 1631  (26.8) | 1530  (26.9) | 1501  (27.9) | 1556  (27.9) | 1470  (28.5) | 1462  (29.1) | 1438  (30.7) | 1402  (29.3) | 1418  (30.8) | 1447  (31.3) | 1355  (31.3) | 1296  (30.3) | 1254  (30.2) | 18760  (29.1) |
| 65 or above (%) | 2385  (39.2) | 2319  (40.7) | 2225  (41.3) | 2328  (41.7) | 2116  (41.0) | 2060  (41.0) | 1863  (39.8) | 1966  (41.1) | 1780  (38.7) | 1926  (41.7) | 1768  (40.8) | 1826  (42.7) | 1826  (44.0) | 26388  (41.0) |
| **Place of birth** | | | | | | | | | | | | | | |
| Hong Kong (%) | 2368  (40.6) | 2122  (39.5) | 2143  (41.6) | 2249  (41.0) | 2009  (40.5) | 1910  (39.3) | 1839  (40.9) | 1803  (39.0) | 1711  (38.1) | 1778  (40.0) | 1678  (39.9) | 1582  (37.1) | 1534  (37.0) | 24726  (39.7) |
| Mainland China and Macau (%) | 3099  (53.2) | 2885  (53.7) | 2656  (51.5) | 2875  (52.4) | 2572  (51.8) | 2571  (52.8) | 2308  (51.3) | 2423  (52.4) | 2330  (51.9) | 2267  (50.9) | 2062  (49.0) | 2065  (48.4) | 1981  (47.8) | 32094  (51.5) |
| Other key Asian countries^1^  (%) | 319  (5.5) | 333  (6.2) | 318  (6.2) | 328  (6.0) | 342  (6.9) | 346  (7.1) | 321  (7.1) | 356  (7.7) | 404  (9.0) | 356  (8.0) | 391  (9.3) | 407  (9.5) | 373  (9.0) | 4594  (7.4) |
| Miscellaneous | 40  (0.7) | 34  (0.6) | 40  (0.8) | 35  (0.6) | 40  (0.8) | 38  (0.8) | 33  (0.7) | 39  (0.8) | 41  (0.9) | 49  (1.1) | 75  (1.8) | 210  (4.9) | 253  (6.1) | 927  (1.5) |
| **Permanent HK residency^2^** | | | | | | | | | | | | | | |
| Yes (%) | 5787  (95.2) | 5403  (94.9) | 5105  (94.9) | 5289  (94.7) | 4856  (94.2) | 4708  (93.7) | 4366  (93.3) | 4413  (92.2) | 4209  (91.6) | 4282  (92.7) | 3917  (91.6) | 3558  (91.0) | 3471  (90.6) | 59364  (93.3) |
| No (%) | 293  (4.8) | 292  (5.1) | 276  (5.1) | 297  (5.3) | 301  (5.8) | 315  (6.3) | 316  (6.7) | 372  (7.8) | 386  (8.4) | 336  (7.3) | 360  (8.4) | 350  (9.0) | 359  (9.4) | 4253  (6.7) |

| **Year** | **2005** | **2006** | **2007** | **2008** | **2009** | **2010** | **2011** | **2012** | **2013** | **2014** | **2015** | **2016** | **2017** | **Total** |
| --- | --- | --- | --- | --- | --- | --- | --- | --- | --- | --- | --- | --- | --- | --- |
| **Employment status** | | | | | | | | | | | | | | |
| Full time employment (%) | 1922  (31.6) | 1767  (31.0) | 1680  (31.2) | 1711  (30.6) | 1682  (32.6) | 1696  (33.8) | 1575  (33.6) | 1602  (33.5) | 1656  (36.0) | 1556  (33.7) | 1455  (34.0) | 1373  (33.9) | 1344  (34.8) | 21019  (32.9) |
| Students (%) | 285  (4.7) | 233  (4.1) | 209  (3.9) | 192  (3.4) | 198  (3.8) | 181  (3.6) | 181  (3.9) | 157  (3.3) | 193  (4.2) | 173  (3.7) | 161  (3.8) | 129  (3.2) | 147  (3.8) | 2439  (3.8) |
| Homemaker (%) | 1024  (16.8) | 926  (16.3) | 914  (17.0) | 892  (16.0) | 775  (15.0) | 717  (14.3) | 571  (12.2) | 593  (12.4) | 597  (13.0) | 452  (9.8) | 477  (11.1) | 493  (12.2) | 439  (11.4) | 8870  (13.9) |
| Retired/ unemployed (%) | 2622  (43.1) | 2594  (45.5) | 2423  (45.0) | 2676  (47.9) | 2403  (46.6) | 2330  (46.4) | 2168  (46.3) | 2185  (45.6) | 1977  (43.0) | 2203  (47.7) | 1945  (45.4) | 1972  (48.6) | 1855  (48.1) | 29353  (46.0) |
| Others (%) | 234  (3.8) | 178  (3.1) | 156  (2.9) | 115  (2.1) | 101  (2.0) | 99  (2.0) | 187  (4.0) | 250  (5.2) | 176  (3.8) | 238  (5.1) | 243  (5.7) | 89  (2.2) | 74  (1.9) | 2140  (3.4) |
| **Case category** | | | | | | | | | | | | | | |
| New cases (%) | 5359  (88.0) | 5048  (88.6) | 4808  (89.3) | 5002  (89.5) | 4622  (89.6) | 4492  (89.4) | 4238  (90.5) | 4323  (90.3) | 4197  (91.3) | 4143  (89.6) | 3984  (93.1) | 3675  (90.7) | 3653  (91.1) | 57544  (90.0) |
| Previous infected/ treated cases (%) | 728  (12.0) | 650  (11.4) | 574  (10.7) | 584  (10.5) | 537  (10.4) | 531  (10.6) | 444  (9.5) | 464  (9.7) | 402  (8.7) | 479  (10.4) | 294  (6.9) | 375  (9.3) | 357  (8.9) | 6419  (10.0) |
| **Disease form (%)** | | | | | | | | | | | | | | |
| Pulmonary TB: with/ without extrapulmonary involvement (%) | 5566  (91.4) | 5189  (91.1) | 4865  (90.4) | 4930  (88.3) | 4495  (87.1) | 4259  (84.8) | 3919  (83.7) | 4016  (83.9) | 3755  (81.6) | 3753  (81.2) | 3410  (78.7) | 3285  (76.9) | 3225  (77.6) | 54667  (84.9) |
| Extrapulmonary TB only (%) | 521  (8.6) | 509  (8.9) | 517  (9.6) | 656  (11.7) | 664  (12.9) | 764  (15.2) | 763  (16.3) | 771  (16.1) | 844  (18.4) | 869  (18.8) | 923  (21.3) | 989  (23.1) | 929  (22.4) | 9719  (15.1) |
| **Smear positive cases** | | | | | | | | | | | | | | |
| Yes (%) | 1813  (31.4) | 1767  (32.9) | 1654  (32.6) | 1634  (31.2) | 1590  (33.4) | 1666  (36.2) | 1550  (36.4) | 1616  (37.0) | 1449  (35.2) | 1349  (33.8) | 1303  (36.6) | 1244  (34.7) | 1113  (34.2) | 19748  (34.1) |
| No (%) | 3955  (68.6) | 3602  (67.1) | 3419  (67.4) | 3604  (68.8) | 3175  (66.6) | 2941  (63.8) | 2703  (63.6) | 2756  (63.0) | 2665  (64.8) | 2648  (66.2) | 2255  (63.4) | 2336  (65.3) | 2143  (65.8) | 38202  (65.9) |
| **Culture confirmed cases** | | | | | | | | | | | | | | |
| Yes (%) | 3936  (69.2) | 3690  (69.1) | 3658  (72.7) | 3424  (69.6) | 2988  (66.7) | 2873  (65.9) | 2676  (66.5) | 2829  (68.1) | 2652  (67.4) | 2695  (70.6) | 2391  (76.0) | 2298  (73.3) | 2274  (75.9) | 38384  (69.8) |
| No (%) | 1753  (30.8) | 1647  (30.9) | 1377  (27.3) | 1493  (30.4) | 1489  (33.3) | 1484  (34.1) | 1347  (33.5) | 1326  (31.9) | 1281  (32.6) | 1120  (29.4) | 754  (24.0) | 838  (26.7) | 722  (24.1) | 16631  (30.2) |
| **Drug resistance cases** | | | | | | | | | | | | | | |
| Yes (%) | 24  (0.4) | 32  (0.6) | 21  (0.4) | 26  (0.5) | 28  (0.5) | 29  (0.6) | 20  (0.4) | 31  (0.6) | 23  (0.5) | 32  (0.7) | 34  (0.8) | 37  (0.9) | 29  (0.7) | 366  (0.6) |
| No (%) | 6063  (99.6) | 5666  (99.4) | 5361  (99.6) | 5560  (99.5) | 5131  (99.5) | 4994  (99.4) | 4662  (99.6) | 4756  (99.4) | 4576  (99.5) | 4590  (99.3) | 4299  (99.2) | 4237  (99.1) | 4125  (99.3) | 64020  (99.4) |

^1^ Including Philippines, Indonesia, Thailand, Nepal, Vietnam, India, Pakistan, and Bangladesh

^2^ 7 years of stay in Hong Kong required for permanent residency, except by birth

**Supplementary Figure 1. Time series plot of monthly age-gender standardised notification rate (SNR) of tuberculosis in Hong Kong from 2005-2017, for a.) male pulmonary tuberculosis (PTB) cases; b.) female PTB cases; c.) male extrapulmonary tuberculosis (EPTB) cases; and d.) female EPTB cases.**

| **a.**  **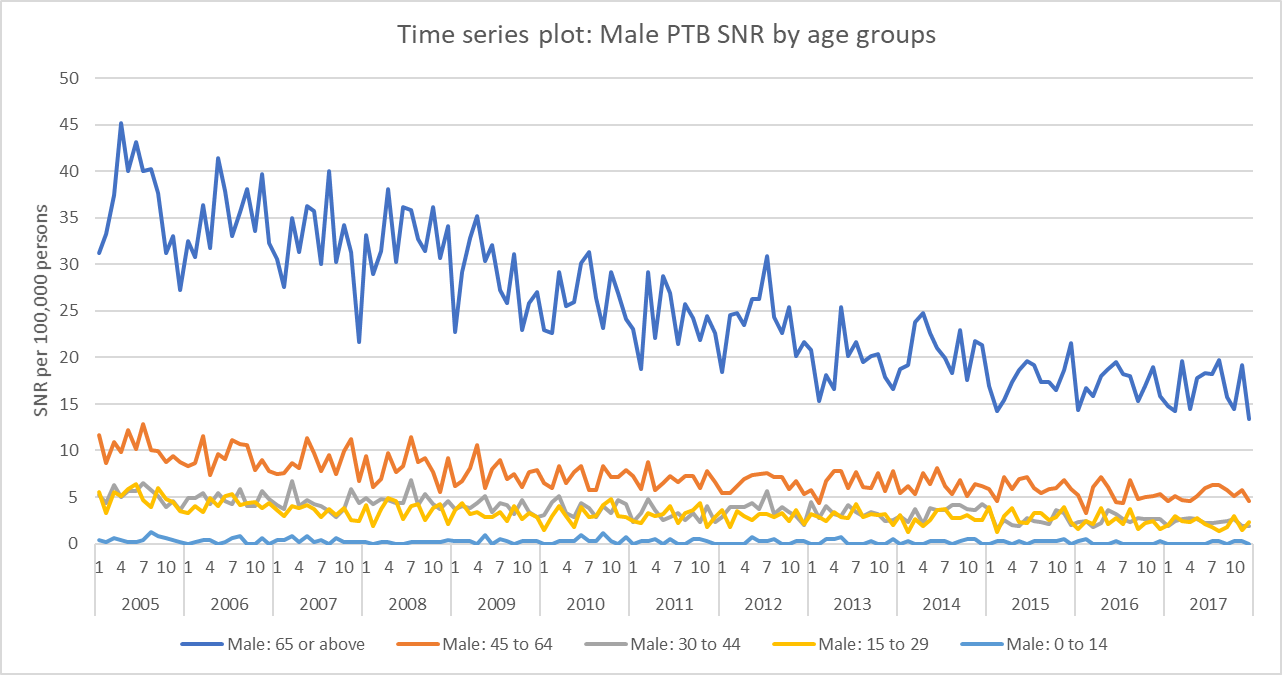** |
| --- |
| **b.**  **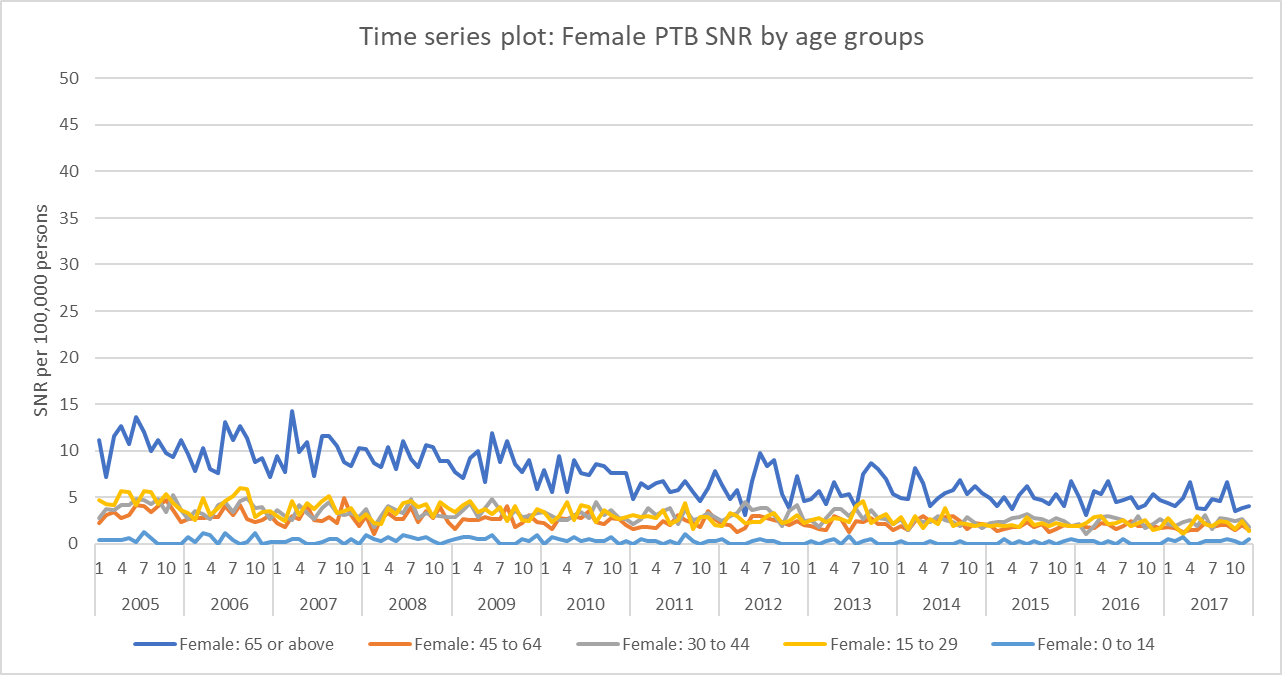** |

| **c.**  **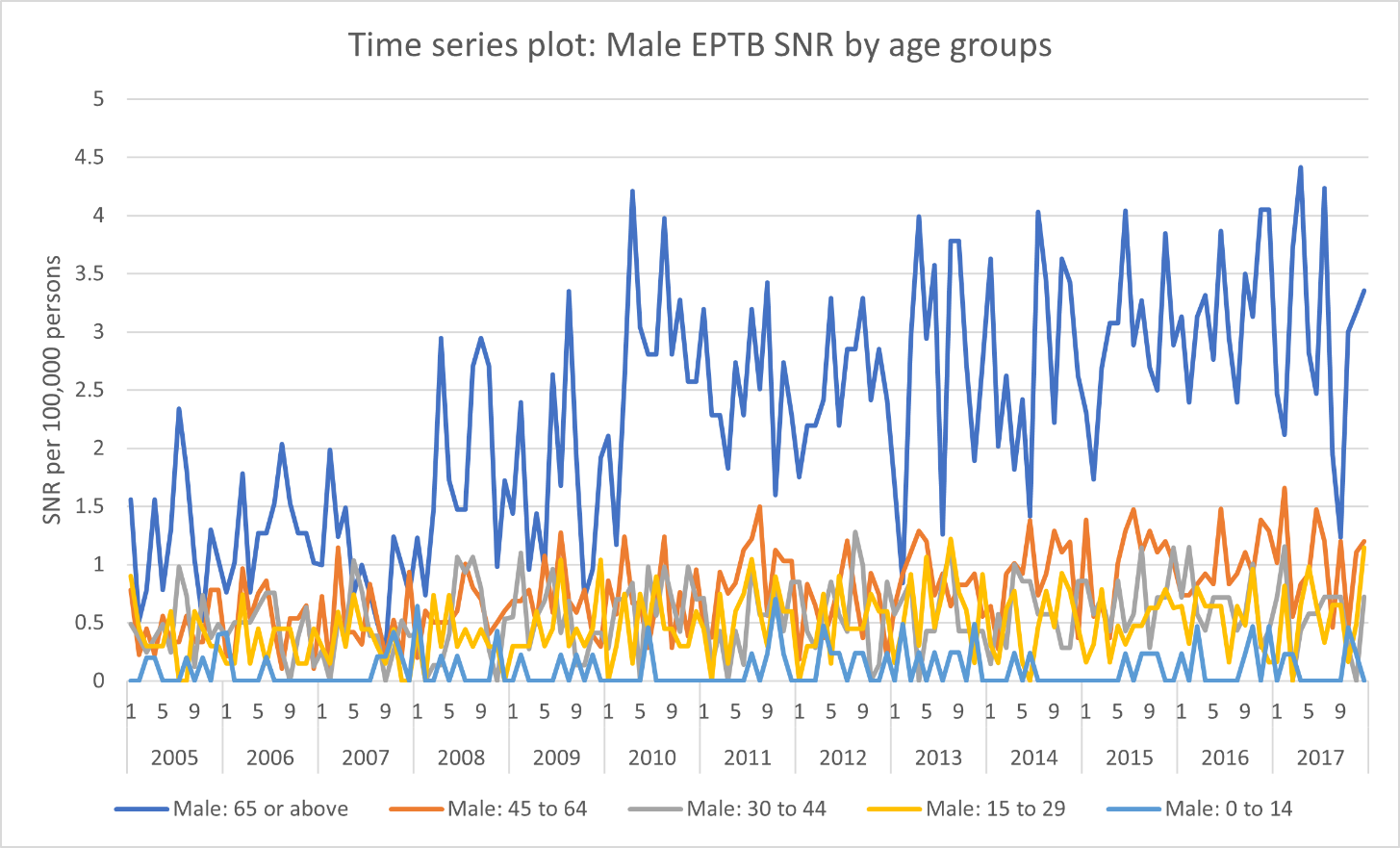** |
| --- |
| **d.**  **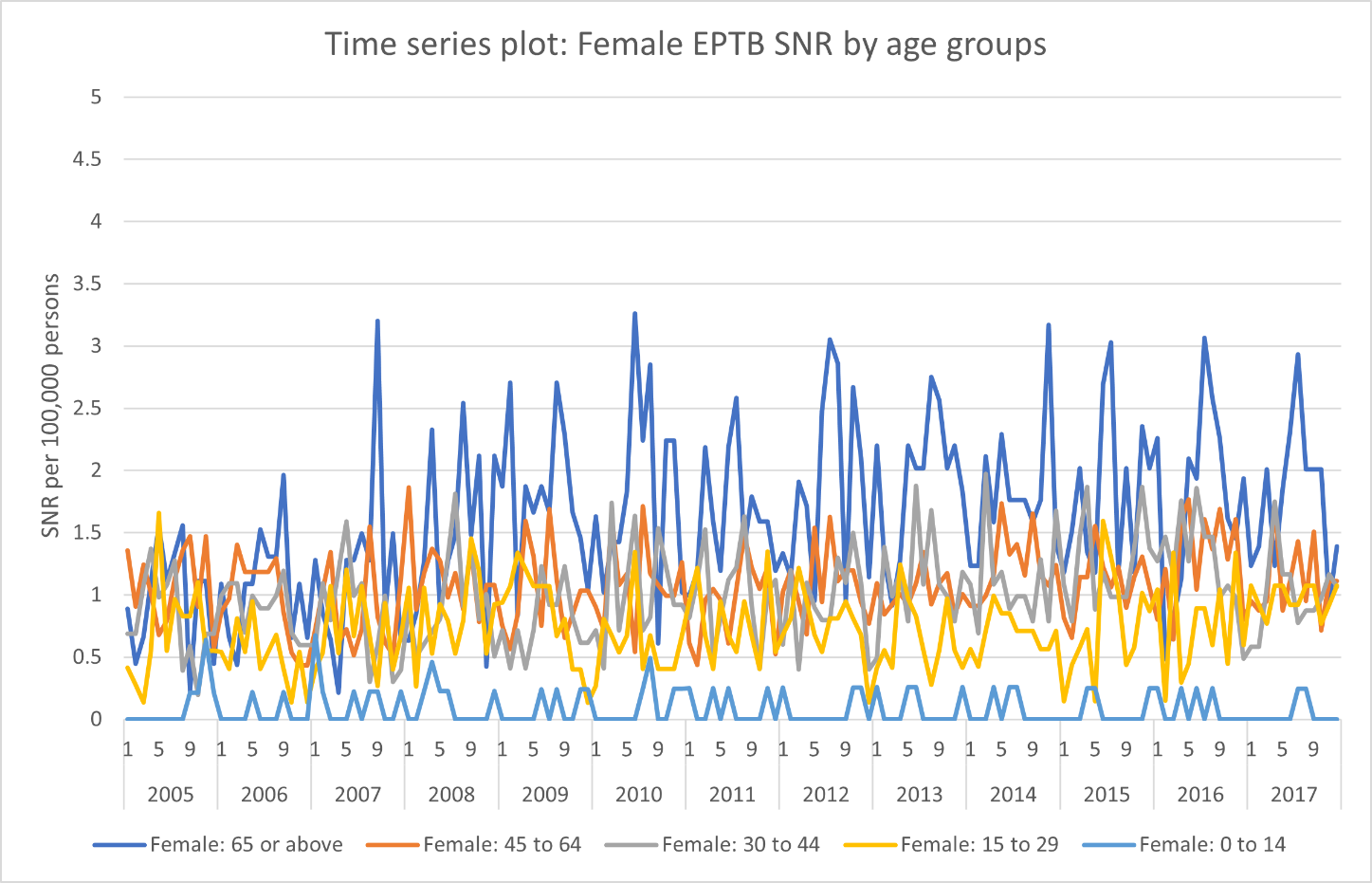** |

**Supplementary Figure 2. Wavelet analysis of the monthly notified TB cases in Hong Kong from 2005-2017 by age and gender, for i.) all forms of TB cases; ii.) pulmonary TB cases; and iii.) extrapulmonary TB cases.** (Right) Global wavelet power spectrum: thick black lines represent the global wavelet power estimates and the grey band indicate the 95% confidence bounds against red-noise background spectra. Significant annual periodicity was indicated when the peak of power exceeding the grey band at one-year period. (Left) Local wavelet power spectrum: Wavelet power value is shown by colour from dark blue (low value) to dark red (high value). Area enclosed with black contour lines indicate the 5% significance level against red noise. Lighter shade area indicates the cone of influence where the edge effect become important and the spectral information is less robust.

| **Male** | | |
| --- | --- | --- |
| **i. All forms Tuberculosis** | **ii. Pulmonary Tuberculosis** | **iii. Extra-pulmonary Tuberculosis** |
| **Elderly (65 or above)** | | |
| 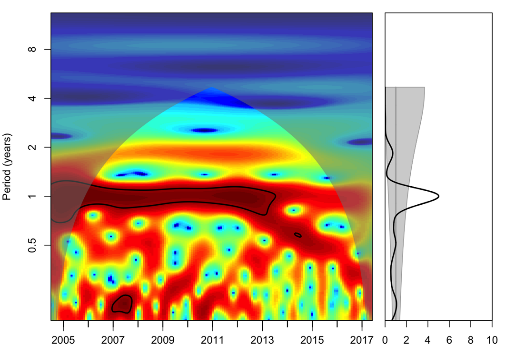 | 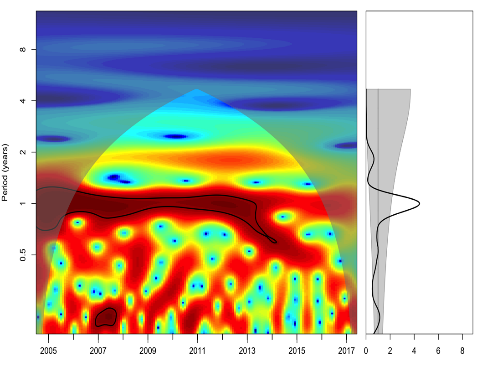 | 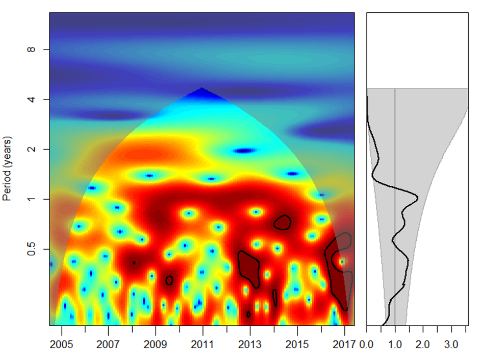 |
| **Middle aged (45-64)** | | |
| 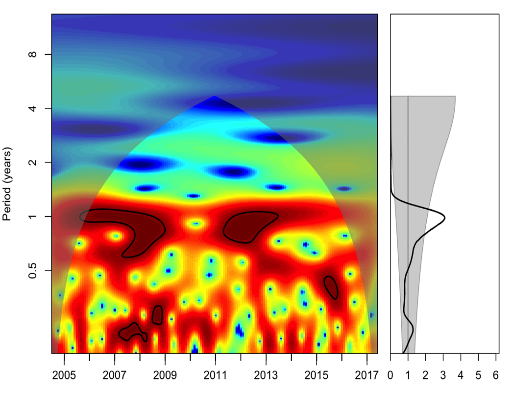 | 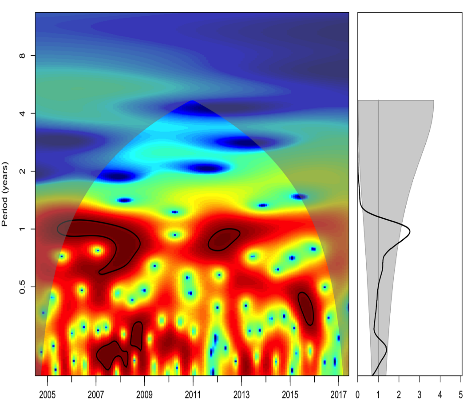 | 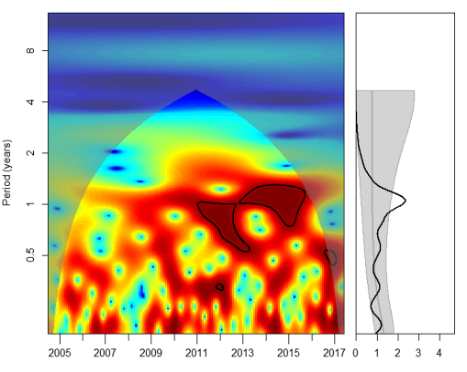 |
| **Adult (30-44)** | | |
| 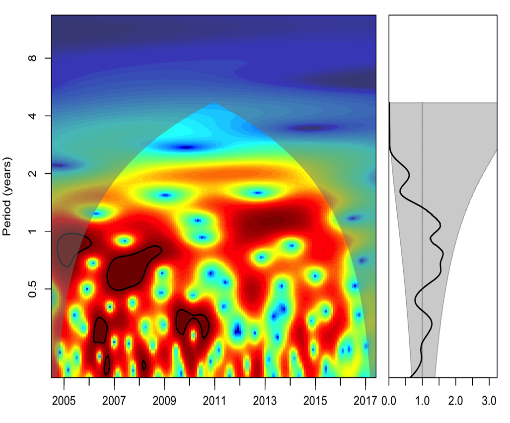 | 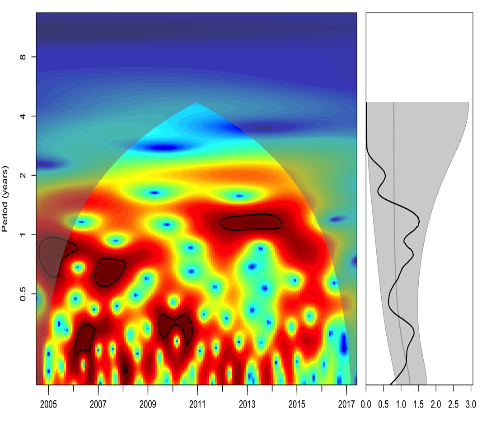 | 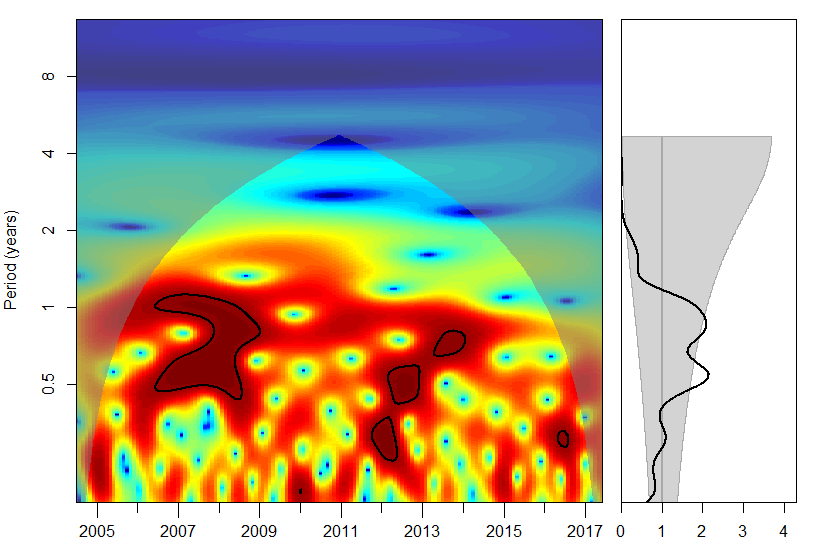 |
| **Adolescents (15-29)** | | |
| 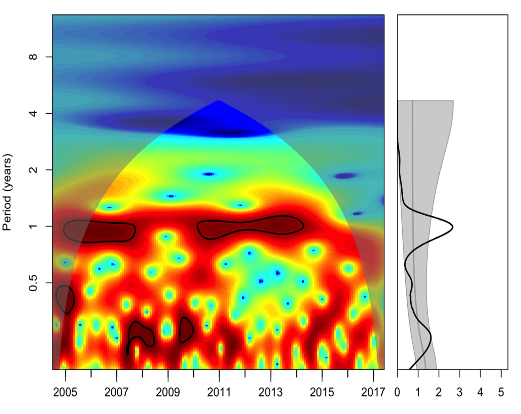 | 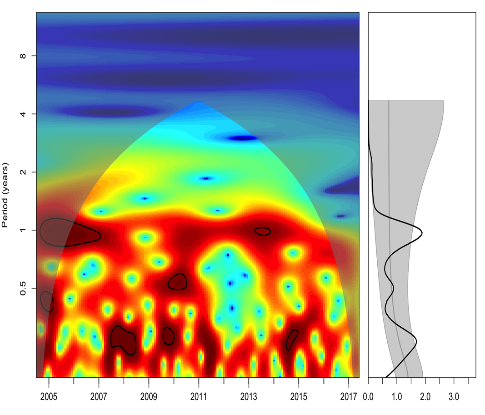 | 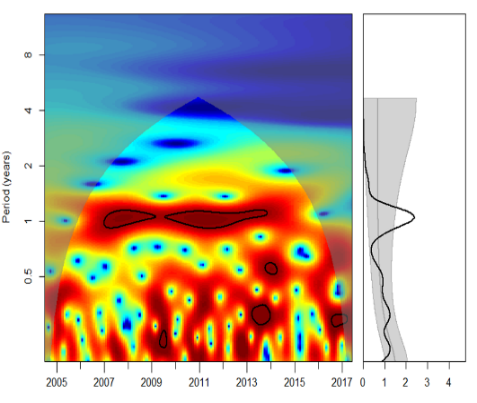 |
| **Children (<14)** | | |
| 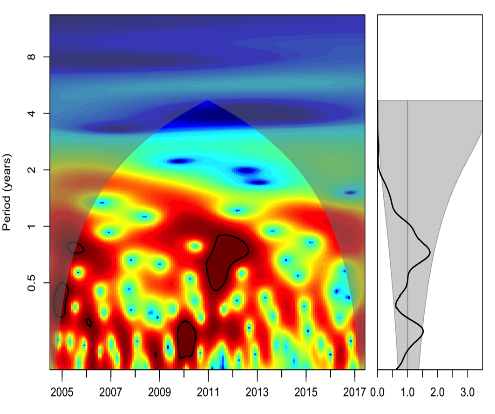 | 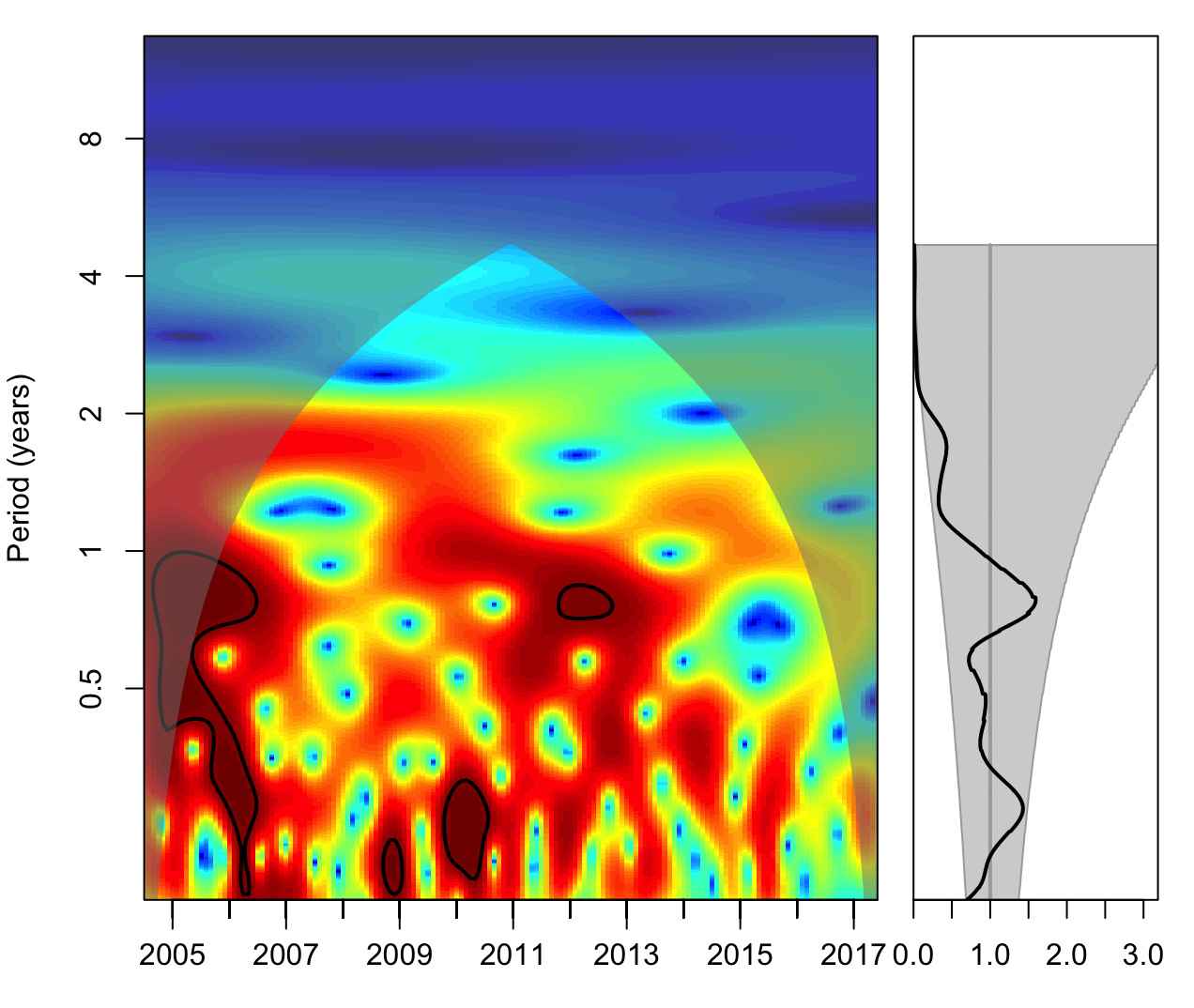 | 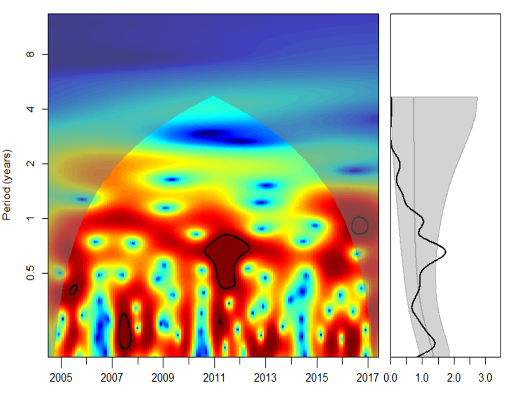 |
| **Female** | | |
| **i. All forms Tuberculosis** | **ii. Pulmonary Tuberculosis** | **iii. Extra-pulmonary Tuberculosis** |
| **Female Elderly (65 or above)** | | |
| **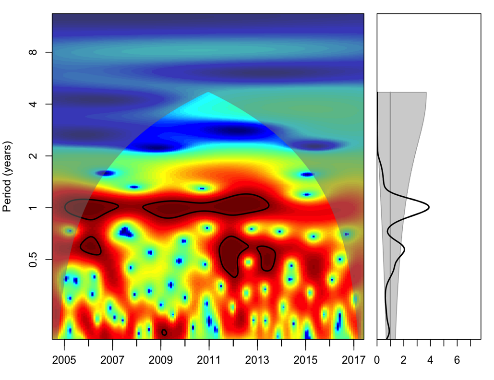** | **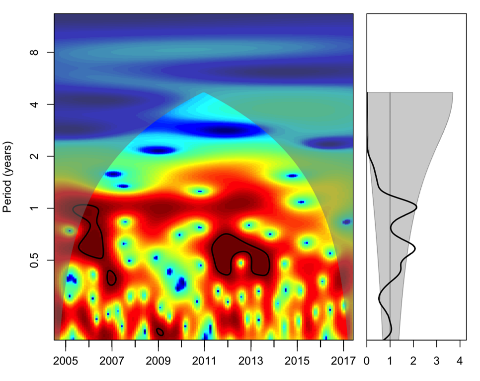** | **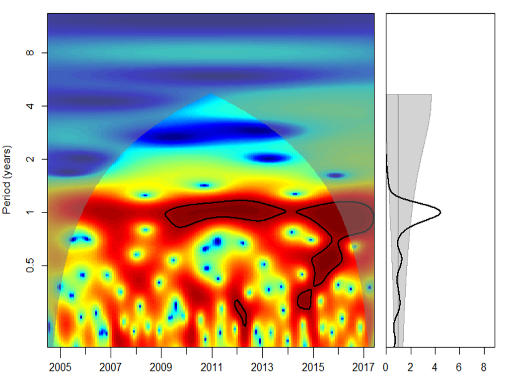** |
| **Female Middle aged (45-64)** | | |
| **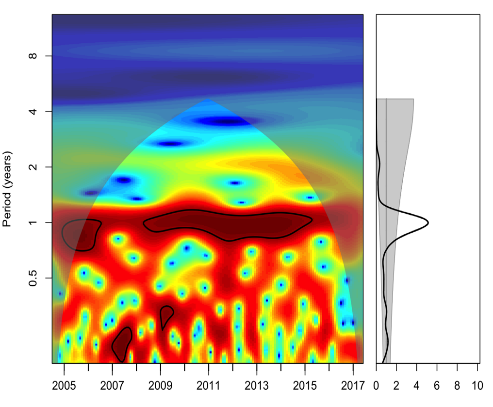** | **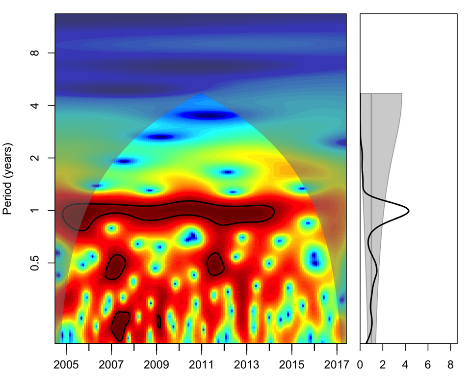** | **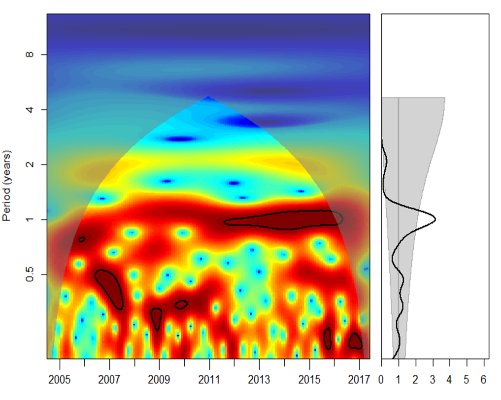** |
| **Female Adult (30-44)** | | |
| **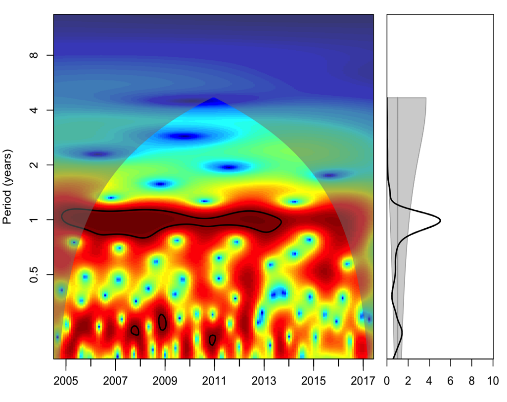** | **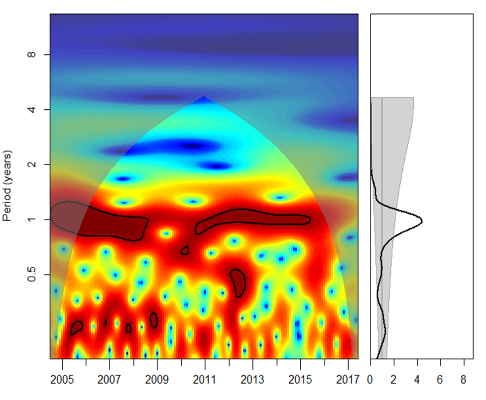** | **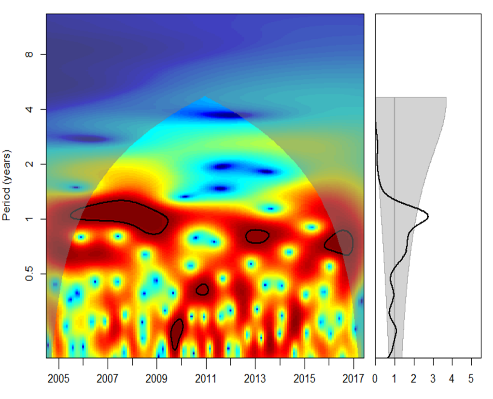** |
| **Female Adolescents (15-29)** | | |
| **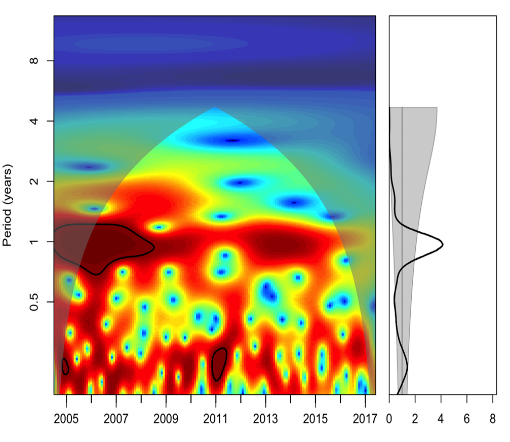** | **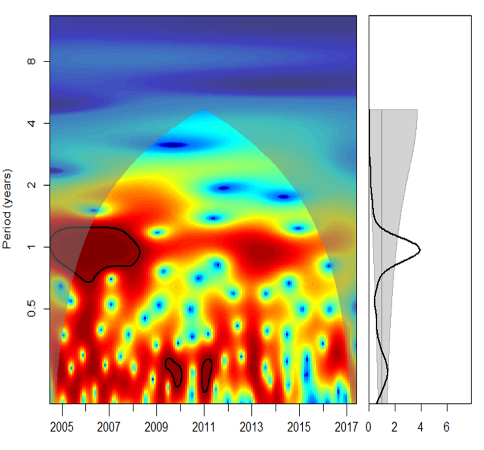** | **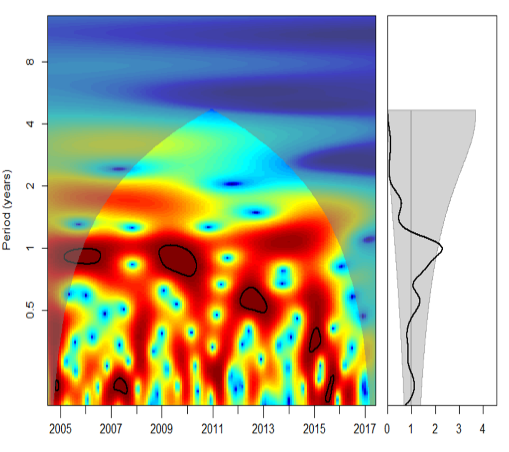** |
| **Female Children (<14)** | | |
| **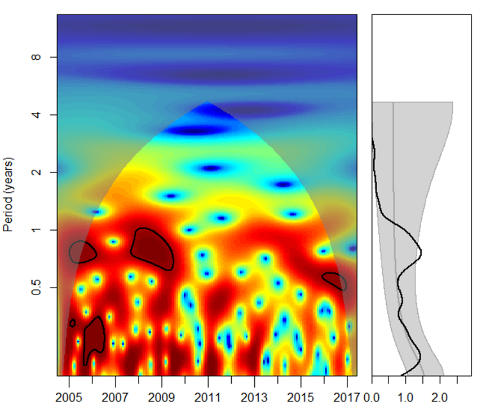** | **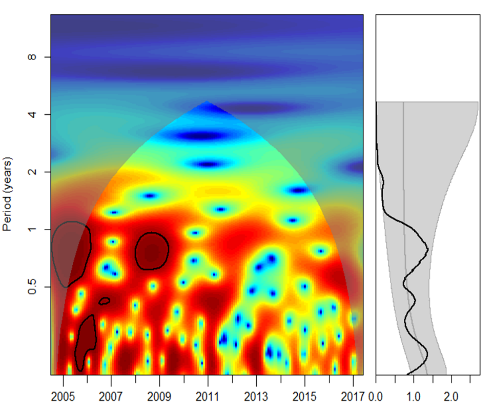** | **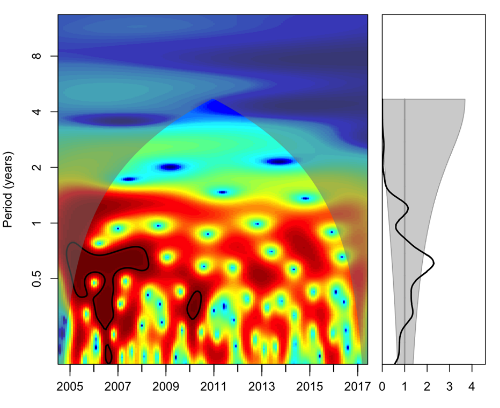** |

**Reference**

1. Cazelles B, Chavez M, Constantin de Magny G, Guégan JF, Hales S. Time-dependent spectral analysis of epidemiological time-series with wavelets. J R Soc Interface. 2007 Aug; 4(15): 625-636.
2. Grinsted A, Moore JC, Jevrejeva S. Application of the cross wavelet transform and wavelet coherence to geophysical time series. Nonlinear Proc Geoph. 2004 Nov; 2004, 11 (5/6), pp.561-566.
3. Christensen AL, Lundbye-Christensen S, Overvad K, Rasmussen LH, Dethlefsen C. Modelling gradually changing seasonal variation in count data using state space models: a cohort study of hospitalization rates of stroke in atrial fibrillation patients in Denmark from 1977 to 2011. BMC Med Res Methodol. 2012 Nov; 12:174.
4. Lundbye-Christensen S, Dethlefsen C, Gorst-Rasmussen A, Fischer T, Schønheyder HC, Rothman KJ, et al. Examining secular trends and seasonality in count data using dynamic generalised linear modelling: a new methodological approach illustrated with hospital discharge data on mycocardial infarction. Eur J Epidemiol. 2009 Mar; 24(5): 225-230.
5. Naumova EN, MacNeill IB. Seasonality assessment for biosurveillance systems. In: Auget JL, Balakrishnan N, Mesbah M, Molenberghs G, editors. Advances in statistical methods for the health sciences. Statistics for industry and technology. Boston, MA : Birkhäuser Boston; 2007. P437-450.
